# ER stress and basement membrane defects combine to cause glomerular and tubular renal disease resulting from *Col4a1* mutations in mice

**DOI:** 10.1242/dmm.021741

**Published:** 2016-02-01

**Authors:** Frances E. Jones, Matthew A. Bailey, Lydia S. Murray, Yinhui Lu, Sarah McNeilly, Ursula Schlötzer-Schrehardt, Rachel Lennon, Yoshikazu Sado, David G. Brownstein, John J. Mullins, Karl E. Kadler, Tom Van Agtmael

**Affiliations:** 1 Institute of Cardiovascular and Medical Sciences, College of Medical, Veterinary and Life Sciences, University of Glasgow, Glasgow, G12 8QQ, UK; 2 British Heart Foundation Centre for Cardiovascular Science, University of Edinburgh, Edinburgh, EH16 4TJ, UK; 3 Wellcome Trust Centre for Cell-Matrix Research, Faculty of Life Sciences, University of Manchester, Manchester M13 9PT, UK; 4 Department of Ophthalmology, University of Erlangen-Nürnberg, D-91054 Erlangen, Germany; 5 Division of Immunology, Shigei Medical Research Institute, Okayama 701-02, Japan; 6 Division of Pathology, School of Molecular and Clinical Medicine, University of Edinburgh, Edinburgh, EH16 4TJ, UK

**Keywords:** Collagen IV, Extracellular matrix, Endoplasmic reticulum stress, Basement membrane, Kidney disease

## Abstract

Collagen IV is a major component of basement membranes, and mutations in *COL4A1*, which encodes collagen IV alpha chain 1, cause a multisystemic disease encompassing cerebrovascular, eye and kidney defects. However, *COL4A1* renal disease remains poorly characterized and its pathomolecular mechanisms are unknown. We show that *Col4a1* mutations in mice cause hypotension and renal disease, including proteinuria and defects in Bowman's capsule and the glomerular basement membrane, indicating a role for Col4a1 in glomerular filtration. Impaired sodium reabsorption in the loop of Henle and distal nephron despite elevated aldosterone levels indicates that tubular defects contribute to the hypotension, highlighting a novel role for the basement membrane in vascular homeostasis by modulation of the tubular response to aldosterone. *Col4a1* mutations also cause diabetes insipidus, whereby the tubular defects lead to polyuria associated with medullary atrophy and a subsequent reduction in the ability to upregulate aquaporin 2 and concentrate urine. Moreover, haematuria, haemorrhage and vascular basement membrane defects confirm an important vascular component. Interestingly, although structural and compositional basement membrane defects occurred in the glomerulus and Bowman's capsule, no tubular basement membrane defects were detected. By contrast, medullary atrophy was associated with chronic ER stress, providing evidence for cell-type-dependent molecular mechanisms of *Col4a1* mutations. These data show that both basement membrane defects and ER stress contribute to *Col4a1* renal disease, which has important implications for the development of treatment strategies for collagenopathies.

## Abstract (precis)

**Summary:** Structural and compositional basement membrane defects and ER stress due to *Col4a1* mutations cause glomerular and tubular kidney disease, and indicate cell-type-specific disease mechanisms for collagen diseases.

## INTRODUCTION

Collagen IV is a major component of the basement membrane (BM), a specialised extracellular matrix structure that provides structural support and influences cell behaviour and signalling. Vertebrates express six collagen IV alpha chains, *Col4a1-Col4a6*, and in the endoplasmic reticulum (ER) three alpha chains form triple-helical protomers that generate three collagen IV networks: α1α1α2(IV), α3α4α5(IV) and α5α5α6(IV) (Khoshnoodi et al., 2008; Van Agtmael and Bruckner-Tuderman, 2010). During kidney development, all BMs contain α1α1α2(IV), whereas in adult kidney it is mainly expressed in vascular, Bowman's capsule, mesangial and tubular BMs (Hudson et al., 2003). The adult glomerular BM (GBM) predominantly contains α3α4α5(IV), and mutations affecting this network cause Alport syndrome (Hudson et al., 2003).

We have previously identified mouse models with *Col4a1* mutations: *Col4a1^+/Raw^* and *Col4a1^+/Svc^*, containing a lysine (K950E) and a glycine substitution (G1064D), respectively (Van Agtmael et al., 2005). Initial histopathological analysis on a mixed genetic background indicated that within this allelic series the glycine mutations appear to cause a more severe phenotype (Van Agtmael et al., 2005). *Col4a1* mouse models are excellent models of the human disease, illustrated by the fact that their analysis led to the identification of humans with *COL4A1*/*COL4A2* mutations (Favor et al., 2007; Gould et al., 2005; Jeanne et al., 2012; Murray et al., 2014; Sibon et al., 2007; Yoneda et al., 2012). Mutations in *COL4A1* and *COL4A2* cause a multisystemic disorder that leads to cerebrovascular disease, eye defects and muscular dystrophy (Vahedi and Alamowitch, 2011). Some patients present with HANAC (hereditary angiopathy, nephropathy, aneurysms and cramps) syndrome and can develop haematuria, Bowman's capsule defects, large renal cysts and reduced glomerular filtration rate (GFR; Plaisier et al., 2007). HANAC syndrome has been proposed as a clinical sub-entity within *COL4A1/COL4A2* disease (Alamowitch et al., 2009), resulting from mutations located in or close to the integrin-binding CB3 region of the collagen protomer predicted to affect integrin signalling (Plaisier et al., 2010). Importantly, *COL4A1/COL4A2* variants have also been implicated in sporadic cases of cerebral vascular disease in the general population (Rannikmae et al., 2015; Weng et al., 2012).

*COL4A1/COL4A2* mutations are associated with BM defects, ER stress and the unfolded protein response (UPR; Gould et al., 2007; Murray et al., 2014; Van Agtmael et al., 2010). ER stress can be induced by the accumulation of misfolded protein within the ER, and the UPR aims to relieve ER stress by reducing general protein synthesis and increasing the levels of chaperones to promote protein folding (Bateman et al., 2009). Although the UPR is a homeostatic response, chronic ER stress activates pro-apoptotic pathways, in part mediated via activation of the protein CHOP (C/EBP homologous protein; Ron and Walter, 2007), and can become pathogenic. Chronic ER stress has recently been implicated in a variety of matrix diseases (Bateman et al., 2009) in addition to kidney diseases such as uromodulin-associated kidney disease (Williams et al., 2009) and Pierson syndrome, caused by mutations in the basement membrane component laminin beta 2 (Chen et al., 2013).

Our initial analysis in *Col4a1* mutant mice revealed a renal component in *Col4a1* disease mainly affecting Bowman's capsule (Van Agtmael et al., 2005), whereas analysis of other mouse models indicated mild proteinuria (Favor et al., 2007). Individuals with HANAC syndrome develop similar defects in Bowman's capsule and also a structural phenotype to the tubular BM and the formation of large cysts (Plaisier et al., 2007), although they do not develop a polycystic kidney disease (Plaisier et al., 2010, 2007). However, the role of this essential BM component in renal pathophysiology remains relatively poorly characterized; for example, the potential progression of *Col4a1* renal disease and its pathomolecular mechanisms are unknown.

Here, we have uncovered that *Col4a1* mutations in mice cause renal glomerular and tubular disease, which becomes more severe with age and leads to proteinuria, polyuria and haematuria. Our data support the suggestion that *Col4a1* mutations can display cell-specific pathomolecular mechanisms, because the glomerular and tubular disease components are associated with BM defects and ER-stress-induced apoptosis, respectively. This has important implications for the development of therapeutic approaches.

## RESULTS

### *Col4a1* renal disease includes renal and tubular disease that develops with age

**Fig. 1.**
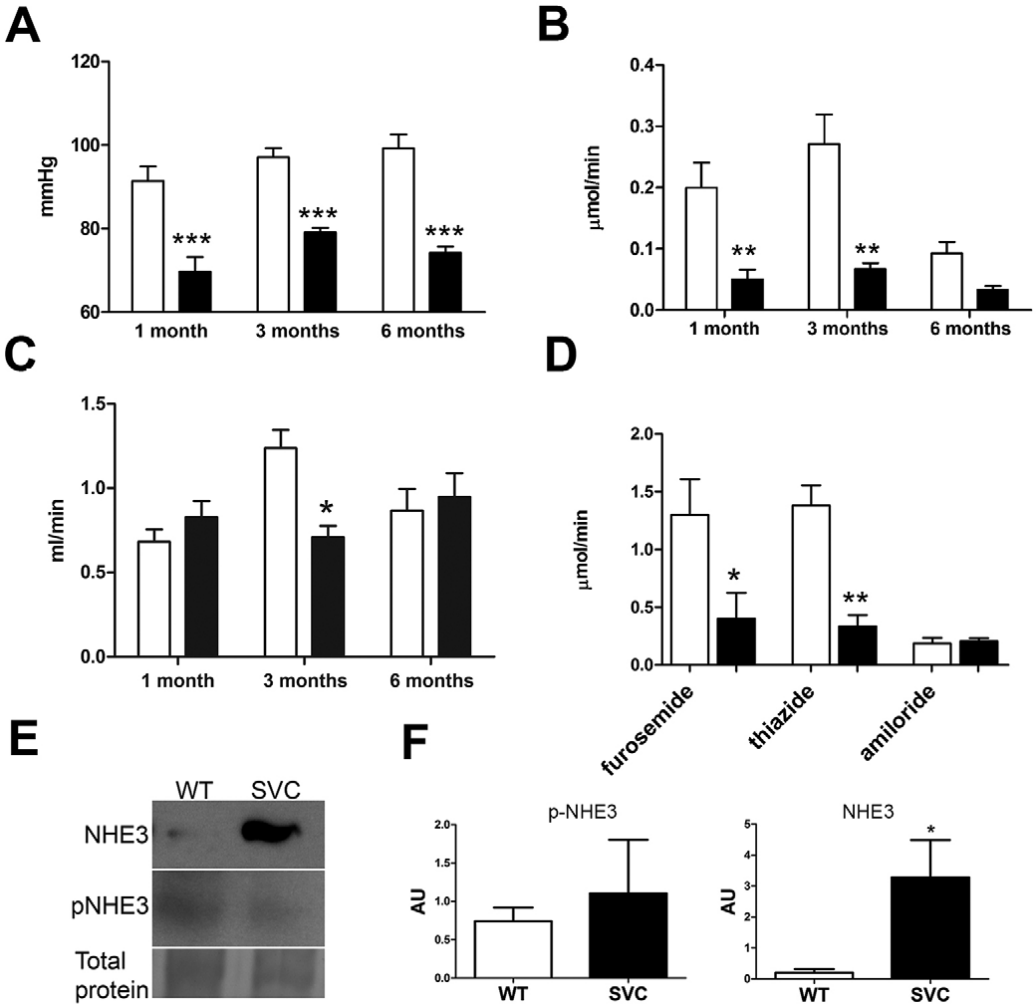
**Analysis of renal function.** (A-D) *In vivo* renal function analysis of 1-, 3- to 4- and 6- to 8-month-old *Col4a1^+/Raw^* (black bars) and WT (white bars) mice. (A) Reduced mean arterial blood pressure in *Col4a1^+/Raw^* animals at all ages. (B) Reduced sodium excretion in *Col4a1^+/Raw^* animals at all ages. (C) Inulin clearance assays uncovered reduced glomerular filtration rate per gram body weight at 3 months (∼40% reduction), but no further decline with age. (D) Measurement of sodium excretion in the presence of the diuretics furosemide and thiazide remains reduced in mutant animals. Blockade of ENaC by amiloride abolished the difference between WT and mutant mice. (E) Western blotting showed ∼16-fold increased levels of total NHE3 (NHE3) in *Col4a1^+/Svc^* (SVC) mice but unaltered phosphorylated NHE3 (p-NHE3). Representative band of total protein stain is given as loading control (entire gel is provided in Fig. S6). (F) ImageJ densitometry analysis of total and p-NHE3. *P<0.05, **P<0.01, ***P<0.001. *n*=5-7 in A-E; *n*=3-5 in F.

We assessed *in vivo* renal function in 3- to 4-month-old *Col4a1^+/Raw^* (Fig. 1; Table 1) and *Col4a1^+/Svc^* (Table 1; Fig. S1) mice, which revealed a reduction in blood pressure of ∼20 mmHg (Fig. 1A; Fig. S1A). *Col4a1^+/Raw^* mice display reduced Na^+^ excretion (Fig. 1B) and GFR as assessed by *in vivo* inulin clearance assays (Fig. 1C; Table 1). *Col4a1* mutant mice have an activated renin-angiotensin system, as indicated by elevated aldosterone levels (Table S1; Van Agtmael et al., 2010). We used diuretic profiling to determine the *in vivo* activity of the major aldosterone-sensitive sodium transport proteins in *Col4a1^+/Raw^* mice (Fig. 1D), whereby responses to furosemide, thiazide and amiloride were measured to assess *in vivo* activity of the Na^+^-K^+^-2Cl^−^ cotransporter (NKCC2), the Na^+^-Cl^−^ cotransporter (NCC) and the epithelial sodium channel (ENaC), respectively (Bailey et al., 2009; Hunter et al., 2014, 2015). Furosemide-sensitive sodium reabsorption was ∼3.5-fold reduced in *Col4a1* mutant mice, but this was a functional downregulation of NKCC2 activity because total protein levels were similar to WT (WT) mice (Fig. S2). Likewise, thiazide-sensitive sodium reabsorption in *Col4a1* mutant mice was approximately fourfold reduced, indicating downregulation of NCC. Amiloride-sensitive sodium reabsorption via ENaC was comparable to WT (Fig. 1D), despite the elevated aldosterone levels. The net increase in tubular sodium reabsorption could be explained by the ∼16-fold increased protein levels of active unphosphorylated sodium hydrogen exchanger (NHE3; Fig. 1E,F).

**Table 1.**
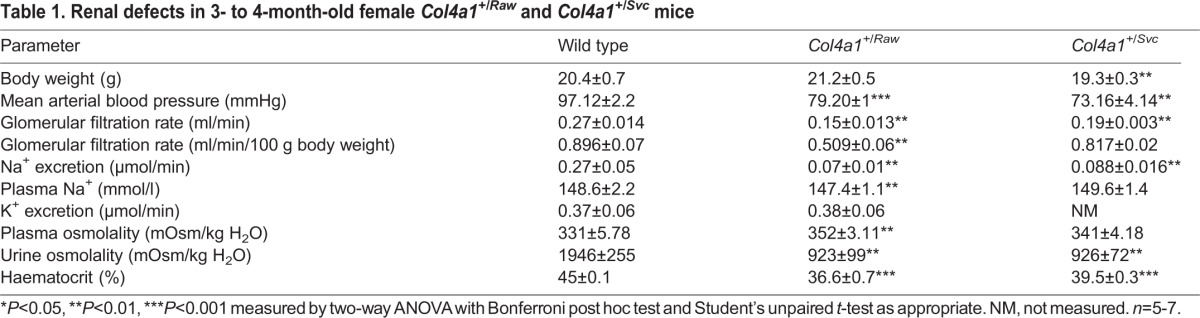
**Renal defects in 3- to 4-month-old female *Col4a1^+/Raw^* and *Col4a1^+/Svc^* mice**

These data indicate that the reduced blood pressure leads to increased sodium reabsorption and activation of the renin-angiotensin-aldosterone system (RAAS) as an attempt to normalize blood pressure. However, the response to the diuretics revealed that *Col4a1* mutant mice have aldosterone insensitivity of the distal nephron, thereby reducing the efficacy of the RAAS to increase blood pressure levels, a factor that could contribute to the development of low blood pressure. To compensate for this deficit, mutant mice activate reabsorption in the proximal tubule via NHE3. A reduction in GFR would reduce the filtered sodium load and also help to preserve sodium homeostasis. However, low GFR was not a consistent feature across the age groups; 1-month-old *Col4a1^+/Raw^* animals revealed a normal GFR and increased sodium reabsorption (Fig. 1B,C; Table S1). We also failed to detect a decline in GFR in 6- to 8-month-old mice (Fig. 1C; Table S2). This absence of a further decline in GFR indicates that *COL4A1* renal disease is unlikely to progress to renal failure, which is supported by a limited reduction of GFR in humans with HANAC syndrome (Plaisier et al., 2007).

### Diabetes insipidus resulting from *Col4a1* mutations

**Fig. 2.**
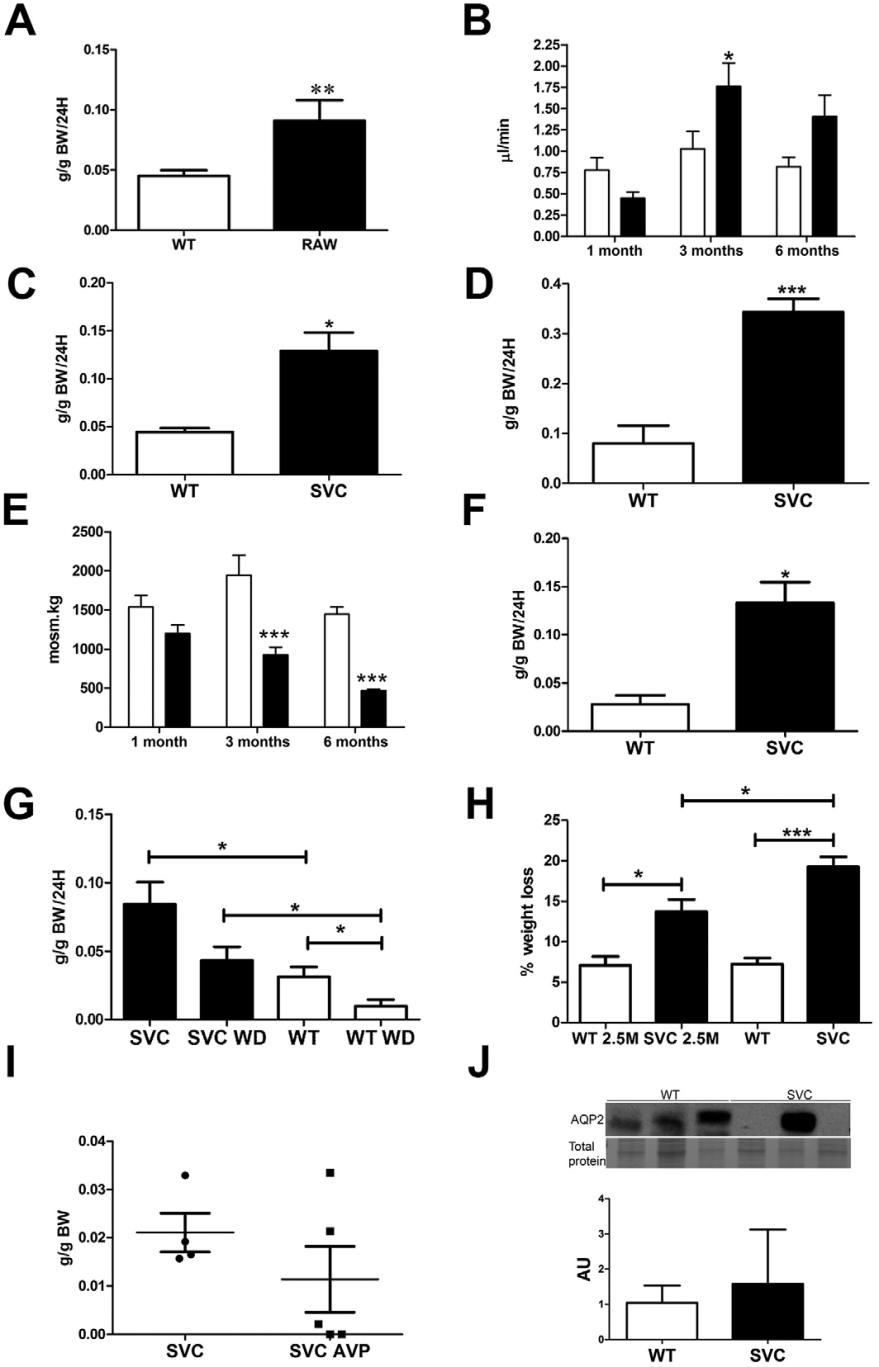
**Diabetes insipidus in *Col4a1* mutant mice.** (A) Metabolic cage study revealed ∼1.8-fold increased 24 h (24H) urine production in *Col4a1^+/Raw^* (RAW) mice per gram body weight (g BW; *n*=6). (B) *In vivo* urine flow was ∼1.7-fold increased in 3- to 4-month-old mice. Older mutant mice displayed similar trends. (*n*=4-6). (C) Metabolic cage study revealed increased daily (24 h) urine production in 4- to 5-month-old *Col4a1^+/Svc^* (SVC) mice per gram body weight (*n*=4-6). (D) Metabolic cage study indicates ∼4.2-fold increased daily water consumption in *Col4a1^+/Svc^* mice (WT, *n*=6; *Col4a1^+/Svc^*, *n*=10). (E) *Col4a1^+/Raw^* mice develop a reduced urine osmolality at 3 months, which becomes more severe with age. Urine osmolality of 3- to 4-month-old *Col4a1^+/Svc^* mice is provided in Fig. S1D (*n*=5-7). (F) Increased urine production in 2.5-month-old *Col4a1^+/Svc^* mice compared with controls (*n*=3). (G) Effect of water deprivation (WD) on urine production in wild-type and *Col4a1^+/Svc^* mice (*n*=4-5). (H) Percentage weight loss following 24 h water deprivation in 2.5- (2.5M) and 4- to 5-month-old animals (2.5M, *n*=3; 4-5 months old, *n*=4-5). (I) Urine production after vasopressin injection. (J) Analysis of Aqp2 protein levels in 4- to 6-month-old *Col4a1^+/Svc^* mice. Representative band of total protein stain is given as loading control (entire gel is provided in Fig. S6) (*n*=3) with densitometry analysis using ImageJ. *P<0.05, **P<0.01, ***P<0.001.

Polyuria and polydipsia are hallmarks of diabetes insipidus that could be attributable to reduced vasopressin production in the brain (central diabetes insipidus) or inability of the kidney to concentrate urine in response to vasopressin (nephrogenic diabetes insipidus; Babey et al., 2011). Despite the low blood pressure and increased sodium reabsorption, a metabolic cage study revealed polyuria in mutant animals, with *Col4a1^+/Raw^* mice displaying a ∼1.8-fold increase in daily urine production (Fig. 2A) and a ∼1.7-fold increase in urine flow rate as measured by *in vivo* renal clearance assays (Fig. 2B), further confirming the tubular disease. Data from *Col4a1^+/Svc^* mice confirmed this (Fig. 2C,D; Table 1; Fig. S1). The polyuria is accompanied by a twofold increase in water consumption (polydipsia; Fig. 2D) and reduced urine osmolality (Fig. 2E; Fig. S1), indicating a defect in concentrating urine that occurs in the distal tubules. Analysis of 1-month-old *Col4a1^+/Raw^* animals revealed normal water excretion and urine osmolality (Fig. 2E; Tables S1 and S2). However, there is a further decline in urine osmolality at 6-8 months, indicating that polyuria becomes more severe with age. Polyuria (∼4.7-fold increase; Fig. 2F) and polydipsia (∼1.6-fold increase; Fig. S1E) were also observed in 2.5-month-old *Col4a1^+/Svc^* mice.

To investigate whether primary polydipsia (increased thirst) causes the apparent diabetes insipidus, mice were deprived of water for 24 h. Although there was an approximately twofold reduction in daily urine output in mutant mice, compared with approximately threefold in controls (Fig. 2G), it remained significantly higher in *Col4a1^+/Svc^* mice. This revealed that mutant mice partly retained the ability to concentrate their urine and excludes primary polydipsia as being causative. Mutant mice had increased weight loss when deprived of water, which became more severe with age (Fig. 2H), indicating dehydration, which combined with the elevated urine output during water deprivation (Fig. 2I), further supports the presence of diabetes insipidus. To distinguish between nephrogenic and central diabetes insipidus, urine output was measured following injection (1 µg/kg) with arginine vasopressin (Gabbi et al., 2012). Overall, 4- to 5-month-old mutant mice maintained an elevated urine output, demonstrating a reduced ability of mutant kidneys to respond to vasopression and thus nephrogenic diabetes insipidus (Fig. 2I). However, a dichotomy was observed, because some mutant mice, which were polyuric, responded to vasopressin, suggesting a component of central diabetes insipidus. Analysis of 2.5-month-old mice revealed increased urine output when deprived of water (Fig. S1F). In accordance with nephrogenic diabetes insipidus, untreated 4-month-old *Col4a1* mutant mice did not have elevated levels of the water channel aquaporin 2 (Aqp2; Fig. 2J) that plays a crucial role in tubular water reabsorption and urine concentration. The variability observed in Aqp2 expression, supported by the vasopressin data, could reflect differing severity of the phenotype, as is characteristic for *Col4a1* mutant mice and patients (Vahedi and Alamowitch, 2011; Van Agtmael et al., 2005), and/or variation in the onset and progression of the defects (see below). Thus, inability to increase Aqp2 expression is in keeping with a generalised homeostatic failure of the distal nephron and, in *Col4a1* mutant mice, leads to a failure to concentrate urine and diabetes insipidus.

### Erythropoietin treatment rescues the hypotension

**Fig. 3.**
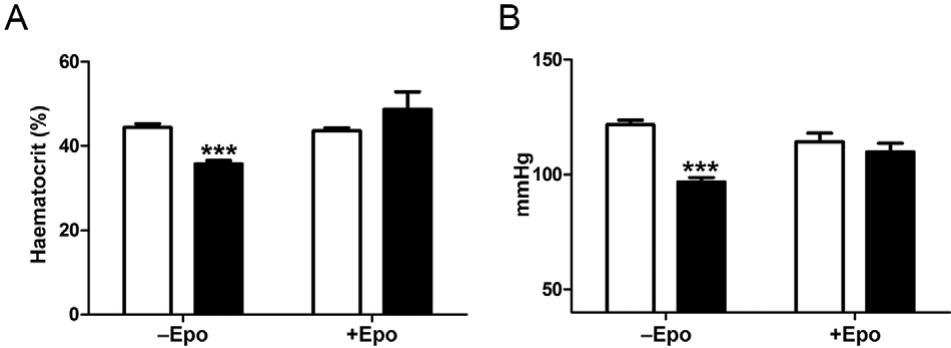
**Erythropoietin treatment of *Col4a1* mutant mice.** (A) Four-month-old *Col4a1^+/Svc^* (black bars) and WT (white bars) mice were treated with Epo, and haematological analysis was performed before (−Epo) and afer Epo (+Epo) treatment, which showed that treatment rescued haematocrit values in *Col4a1^+/Svc^* mice (WT 44% pretreatment, 44% post-treatment; *Col4a1^+/Svc^* 36% pre-treatment, 49% post-treatment; *n*=4-5). (B) Tail-cuff plethysmography revealed a rescue of blood pressure in Epo-treated mice (WT 122 mmHg pre-treatment, 114 mmHg post-treatment; *Col4a1^+/Svc^* 97 mmHg pre-treatment, 110 mmHg post-treatment; *n*=4-5). *P<0.05, **P<0.01, ***P<0.001, Student's unpaired *t*-test.

*Col4a1* mutant mice display reduced blood volume and haematocrit levels but normal plasma volume (Van Agtmael et al., 2010), and recently, anaemia has been described in individuals with a *COL4A1* mutation (Tomotaki et al., 2014; Yoneda et al., 2013). The mechanism underlying the reduced red blood cell number remained unexplored, but a defect in erythropoiesis or erythrocyte maturation could be a contributing factor whereby the kidney disease affects renal erythropoietin production. To determine whether the reduced haematocrit in *Col4a1* mutant mice can be explained by reduced erythrocyte maturation, *Col4a1^+/Svc^* mice were treated with human recombinant erythropoietin (Epo) for 2 weeks. Interestingly, Epo treatment rescued haematocrit levels in 4-month-old *Col4a1^+/Svc^* mice (Fig. 3A) and the increased haematocrit levels were associated with an increased in blood pressure in *Col4a1* mutant mice (Fig. 3B). These data confirm that reduced renal Epo production and subsequent erythrocyte maturation contribute to the haematological and blood pressure phenotypes.

### Age-dependent atrophy of the medulla and Bowman's capsule defects contribute to renal dysfunction

**Fig. 4.**
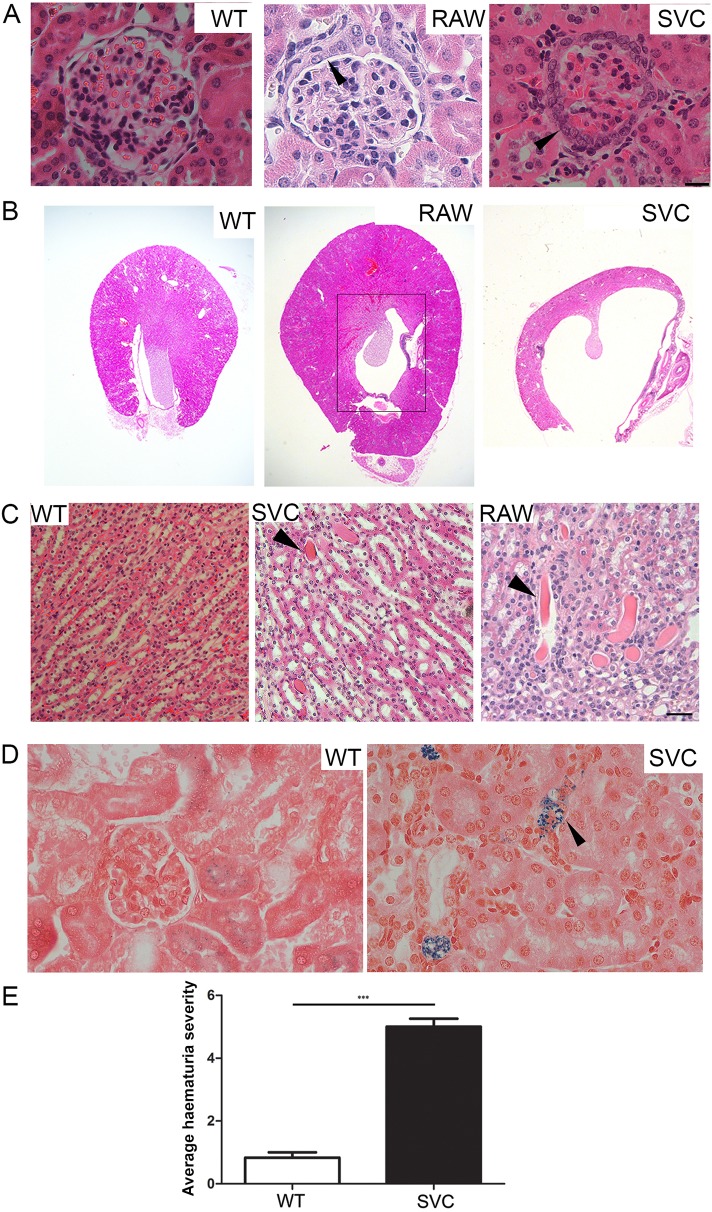
**Analysis of kidney histopathology.** (A) Bowman's capsule defects in 3- to 4-month-old *Col4a1^+/Raw^* (RAW) and *Col4a1^+/Svc^* (SVC) mice include thickening of the capsule (arrowhead) and formation of multiple cell layers of parietal epithelial cells, which can develop a more cuboid appearance. (B) Haematoxylin and Eosin staining revealed atrophy of the medulla (indicated by black box) in 8-month-old *Col4a1^+/Raw^* and *Col4a1^+/Svc^* mice that develops in adulthood (see Table S3). (C) Protein cast (arrowhead) in kidney sections of *Col4a1^+/Raw^* and *Col4a1^+/Svc^* mice (*n*=3, 6 months old). (D) Perls' Prussian Blue staining of renal sections identified haem deposits (arrowhead) in 4-month-old *Col4a1^+/Svc^* mice as a sign of haemorrhage. Perls' Prussian Blue staining does not detect intact red blood cells (*n*=3; scale bars: 20 µm). (E) Semi-quantitative analysis of urine samples using a dipstick revealed haematuria in mutant mice. Absence of haematuria was set as a value of 1 (see Fig. S4C for haematuria scale; *n*=6). ***P<0.001.

To determine any morphological basis to the renal dysfunction, we performed Haematoxylin and Eosin staining on 21- and 40-day-old, 3- to 4- and 6- to 8-month-old *Col4a1^+/Raw^* mice (Fig. 4A,B; Table S3). This identified glomerulopathy characterized by hypertrophy and a more cuboidal appearance of the parietal epithelium of Bowman's capsule (Van Agtmael et al., 2005; Fig. 4A), which develops fully by 40 days of age (Table S3). No significant difference in cellularity of the glomerulus or glomerulosclerosis was detected, confirming that the glomerulopathy primarily affects Bowman's capsule. The morphological defects progress with age, because at around 3 months atrophy of the medulla begins to develop and becomes fully penetrant by 6 months (Fig. 4B; Table S3). Hydronephrosis might also contribute to the atrophy of the medulla, because mutant animals develop distended bladders (data not shown). However, mutant mice urinate freely as indicated by our metabolic cage studies, excluding an obstruction in the urethra being the major cause. These data were confirmed in 40-day-old and 3- to 4-month-old *Col4a1^+/Svc^* mice (Fig. 4A,B and data not shown). The onset of atrophy of the medulla corresponds with the increased urine flow rate, and further reduced urine osmolality (Fig. 2E). This suggests that the medullary atrophy is likely to prevent the ability to increase Aqp2 levels sufficiently to concentrate the urine, resulting in a nephrogenic component to the diabetes insipidus. Combined with the data showing polyuria in 2.5-month-old animals (Fig. 2F), which is before the medullary atrophy has developed, this provides evidence for the development of an initial central diabetes insipidus with an additional nephrogenic component as animals age.

To investigate the development of any fibrosis, we performed PicroSirius Red staining and immunostaining against collagen I. Although increased collagen I staining could occasionally be observed in the perivascular tissue of some blood vessels in mutant kidneys, this analysis failed to detect any signs of overt renal fibrosis (Fig. S3A). Finally, absence of renal phenotypes in 3-month-old *Col4a1^+/Raw^* mice on a mixed genetic background (Van Agtmael et al., 2005) confirms that genetic modifiers influence *Col4a1* renal disease.

### *Col4a1* mutant mice develop proteinuria and haematuria

Although Col4a1 is not major component of the GBM, histopathological analysis of 3- to 4-month-old mice revealed protein casts within tubules (Fig. 4C), suggesting the development of proteinuria. This is supported by increased total protein levels in *Col4a1^+/Raw^* and *Col4a1^+/Svc^* urine samples analysed by SDS-PAGE (Fig. S4A). Enzyme-linked immunosorbent assay (ELISA) analysis was performed to measure albumin-to-creatinine ratios of urines samples, which revealed increased albumin-to-creatinine ratios in mutant mice (Fig. S4B), confirming the development of mild proteinuria and highlighting an under-recognised role for α1α1α2(IV) in the filtration apparatus. Besides polyuria and proteinuria, mice also develop haematuria (Fig. 4D,E), similar to humans with HANAC syndrome (Plaisier et al., 2010, 2007), as indicated by dipstick analysis of urine samples collected in metabolic cage studies [*Col4a1^+/Svc^* (6/6) *Col4a1^+/Raw^* (1/8) and wild-type (0/10); Fig. 4E]. Perls staining of kidney sections revealed haem deposits in renal tissue (Fig. 4D), confirming that renal haemorrhage underlies the haematuria and that vascular defects contribute to renal pathophysiology.

### *Col4a1* mutations affect BM structure and composition

**Fig. 5.**
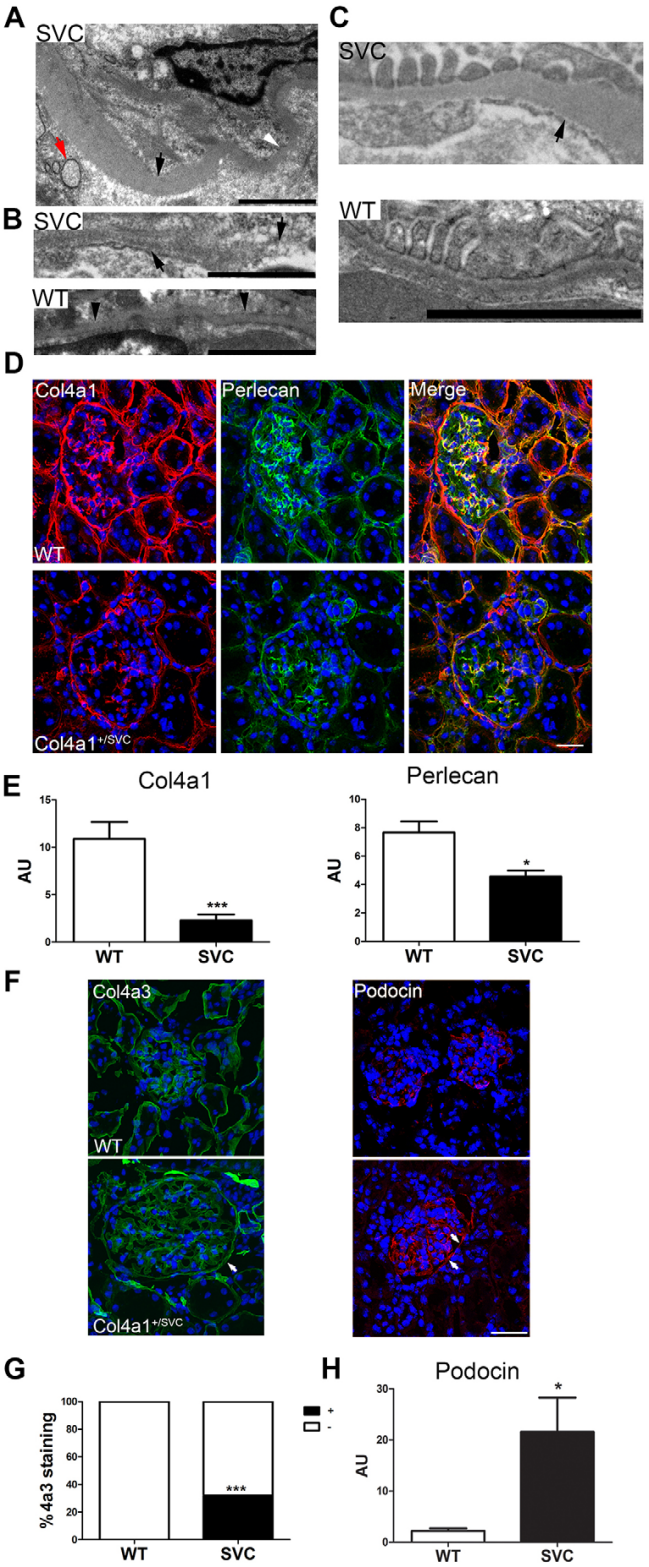
**Investigation of mechanisms underlying *Col4a1* kidney disease.** (A) Transmission electron microscopic analysis revealed irregular thickening of the BM of 4-month-old *Col4a1^+/Svc^* Bowman's capsule (indicated by black arrowhead; white arrowhead indicates thinner region). The presence of swollen ER vesicles can also be observed (red arrow). (B) The vascular BM in 4-month-old *Col4a1^+/Svc^* mice (SVC) displayed focal interruptions (right black arrow) and an irregular less dense appearance with BM fragments (left arrow). (C) The GBM in 4-month-old *Col4a1^+/Svc^* mice showed irregular thickening (black arrow), but no interruptions were detected. Scale bars: 2 µm in A-C (*n*=3). (D) Immunostaining against Col4a1 and perlecan in 4-month-old *Col4a1^+/Svc^* kidney sections followed by confocal microscopy revealed reduced deposition of Col4a1 and perlecan in BMs of *Col4a1^+/Svc^* mice (*n*=3). Scale bar: 20 μm. (E) ImageJ analysis of fluorescence staining in D. (F) Immunostaining against Col4a3 (green) and podocin (red) revealed deposition in 3- to 4-month-old mutant BM of Bowmans capsule (white arrows). Scale bar: 20 µm. (G) Fraction of Bowman's capsules that stain positive (black) and negative (white) for Col4a3 in WT (0% positive) and *Col4a1^+/Svc^* mice (32% positive; *n*=85 glomeruli across three animals, Fisher's exact test). (H) ImageJ analysis of podocin fluorescence staining in H revealed ∼9.8-fold increase in SVC. *P<0.05, ***P<0.001, Student's unpaired *t*-test in E,H; Fisher's exact test in G.

To shed light on pathomolecular disease mechanisms, electron microscopy was performed on 3- to 4-month-old mice, which revealed structural BM defects predominantly in the BMs of Bowman's capsule and the vasculature (Fig. 5A-C; Fig. S3C). The BM of Bowman's capsule revealed irregular thickening (Fig. 5A) and, in some instances, a basket-weave appearance was observed. Vascular BMs displayed extensive defects, including fragmentation and focal absence (Fig. 5B), likely causing vascular fragility, haemorrhage and haematuria, underscoring the vascular component of *Col4a1* renal disease. Analysis of the GBM revealed irregular thickening, although no interruptions or thinning was observed (Fig. 5C). The presence of these defects and proteinuria indicates a role for α1α1α2(IV) in the filtration apparatus. No structural defects were observed in BM of tubules (Fig. S3C).

To investigate whether the BM defects are the result, at least in part, of reduced α1α1α2(IV) incorporation, we performed immunohistochemistry against Col4a1. This revealed a ∼80% reduction in Col4a1 staining in tubular, mesangial and Bowman's capsule BMs (Fig. 5D,E; Fig. S3B). The reduced α1α1α2(IV) deposition was accompanied by ∼40% reduction in perlecan staining in all major BMs (Fig. 5D,E), but no consistent differences were observed for laminin or nidogen 1 and 2 (Fig. S5). Interestingly, ∼32% of mutant Bowman's capsules stained positive for Col4a3, compared with 0% for WT, confirming ectopic deposition of α3α4α5(IV) in damaged BMs of Bowman's capsule (Fig. 5F,G). This ectopic expression could reflect that mutant parietal epithelial cells express different collagen networks and/or that these cells have adopted a more ‘podocyte cell-like’ nature, because Col4a3 is normally produced by podocytes (Abrahamson et al., 2009). Immunostaining against podocin (marker for podocytes) detected podocin staining in Bowman's capsule of mutant mice, suggesting that at least some parietal epithelial cells might have undergone cell reprogramming. Furthermore, a ∼9.8-fold increase in podocin staining was observed within the glomerulus (Fig. 5F,H). These data indicate that *Col4a1* mutations not only affect BM structure but also BM composition.

### Chronic ER stress in *Col4a1* renal disease

**Fig. 6.**
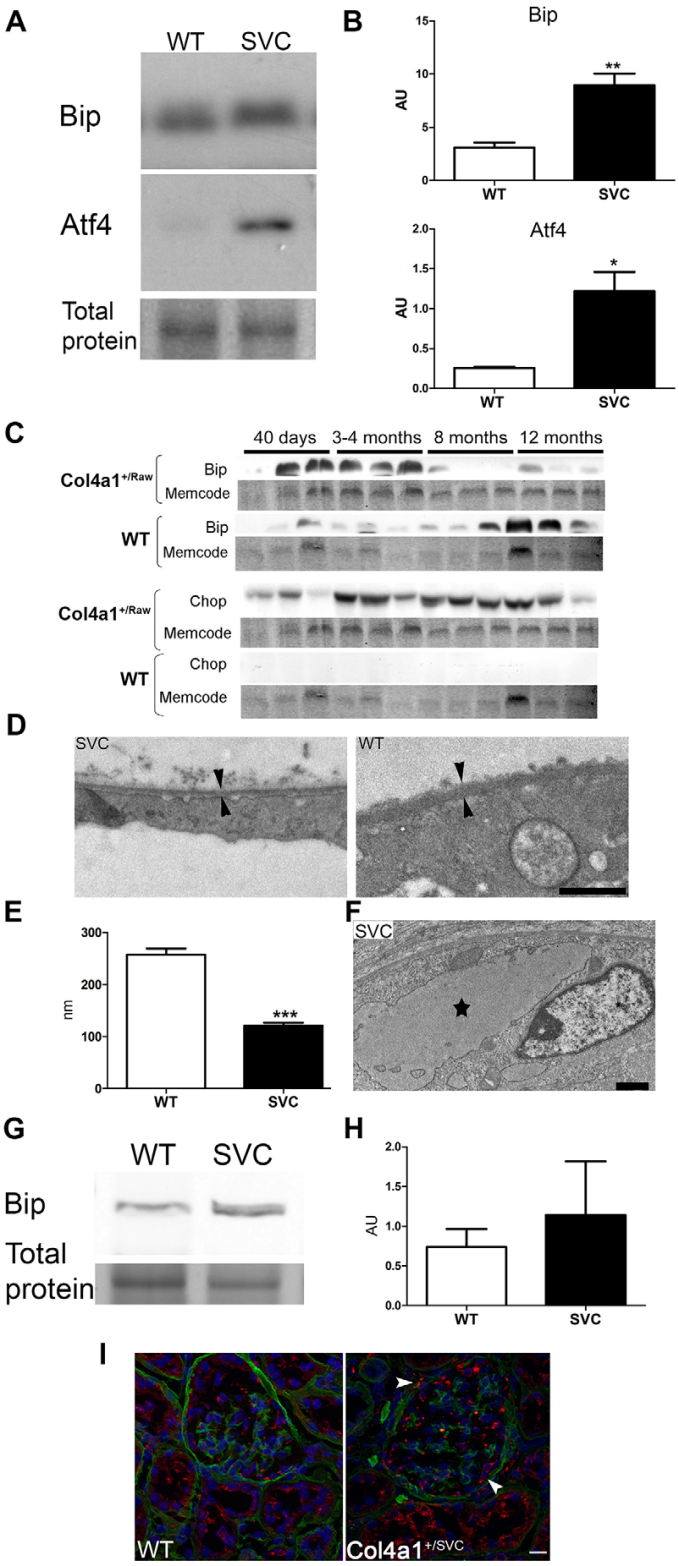
**ER stress and renal disease.** (A) Significantly increased protein levels of ER stress markers Bip (∼2.9-fold increase) and Atf4 (∼4.6-fold increase) in 4-month-old *Col4a1^+/Svc^* mice (*n*=3)*.* Representative band of total protein stain is given as loading control (entire gel is provided in Fig. S6). (B) Densitometric analysis of Bip and Atf4 using ImageJ. (C) Examination of protein levels of Bip and CHOP analysis across *Col4a1^+/Raw^* animals of different ages (40 days, 3-4, 8 and 12 months). Increased protein levels of Bip in *Col4a1^+/Raw^* animals precedes elevated levels of CHOP, which is a marker for chronic ER-stress-induced apoptosis. Activation of CHOP coincides with development of atrophy of the medulla (see Table S3; *n*=3). (D) *Col4a1^+/Svc^* mice display thinning of the BM of Bowman's capsule at 40 days of age. Scale bar: 2 μm. (E) Average BM thickness as measured by ImageJ (WT ∼257 nm; *Col4a1^+/Svc^* ∼121 nm; *n*=3). (F) Distended ER vesicle (black star) in epithelial cell of Bowman's capsule in 40-day-old *Col4a1^+/Svc^*. Scale bar: 2 μm. (G) Analysis of Bip protein levels in 40-day-old *Col4a1^+/Svc^* mice (*n*=3)*.* Representative band of total protein stain is given as loading control (entire gel is provided in Fig. S6). (H) Densitometric analysis of G. (I) Immunostaining against Bip (red) and Col4a1 (green) on kidneys of 3-month-old mice revealed increased expression in epithelial cell of Bowman's capsule (white arrow), and apparent more intense staining in *Col4a1^+/Svc^*. Scale bar: 20 µm (*n*=3).

Electron microscopic analysis revealed enlarged ER vesicles (Fig. 5A; Fig. 6F) in epithelial cells of Bowman's capsule of mutant mice, suggestive of ER stress. Activation of ER stress was confirmed by western blotting in kidneys of 3- to 4-month-old mutant mice, which had a ∼2.9- and ∼3.6-fold increase in protein levels of the ER stress markers Bip and Atf4, respectively (Fig. 6A,B).

To shed light on the relative contribution of ER stress and BM defects to the renal pathology, we investigated the association of BM defects and ER stress with the development of renal defects in the *Col4a1^+/Raw^* cohorts used to establish phenotype progression (Table S3). Western blot analysis against Bip revealed an apparent transient increase (Fig. 6C) in younger mice, followed by marked elevation of CHOP at time points that appear to correspond to development of medullary atrophy (Fig. 6C). In the UPR, CHOP is downstream of Bip, reflects progression to chronic pathogenic ER stress and is associated with ER-associated apoptosis (Bateman et al., 2009; Ron and Walter, 2007). All 3- to 4-month-old *Col4a1^+/Raw^* mice also expressed cleaved caspase 3 (Fig. S6E). Interestingly, although chronic ER stress coincides with development of medullary atrophy, no significant BM abnormalities were observed in tubules (Fig. S3), strongly arguing for a causative role of chronic ER stress in renal tubular disease. By contrast, the glomerulopathy becomes apparent at 21-40 days of age and is associated with BM defects but before the onset of the robust elevation of CHOP levels. Likewise, 40-day-old *Col4a1^+/Svc^* kidneys have a ∼50% reduction in thickness of the BM in Bowman's capsule (Fig. 6D,E). Intriguingly, a distended ER could be observed in some parietal epithelial cells of Bowman's capsule (Fig. 6F). However, although this indicates ER retention of collagen IV and suggests ER stress in some cells, this did not translate into higher ER stress levels at a tissue level as we failed to detect elevated Bip protein levels (Fig. 6G). By contrast, significantly elevated Bip levels were detected in protein homogenates of kidneys at 3-4 months (Fig. 6A,B). This was confirmed by immunostaining against Bip on kidney sections of 3-month-old mutant mice, which identified Bip expression in the epithelial cells of Bowman's capsule (Fig. 6I). In some tubules and the mesangium, a more punctate intense staining was also observed (Fig. 6I).

Overall, these data lend support to an adaptive ER stress response in glomeruli and a chronic pathogenic response in tubules. Interestingly and importantly, this differential response has also been observed after pharmacological induction of ER stress (Inagi, 2009). Therefore, our data lend support to a disease mechanism with variable relative contributions of ER stress and BM defects, whereby BM defects play a major role in the glomerulopathy and ER-stress-induced apoptosis in the tubulopathy.

## DISCUSSION

Our analysis of the development and progression of renal disease caused by *Col4a1* mutations has expanded the spectrum of potential clinical defects that can develop in humans with *COL4A1* mutations and identified the importance of α1α1α2(IV) in adult kidney function and pathophysiology. *Col4a1* mutations cause a glomerulopathy and tubulopathy with medullary atrophy, leading to proteinuria and diabetes insipidus because of a defect in the ability to concentrate urine. The haematuria attributable to haemorrhage illustrates an important vascular component to the renal disease. The progressive nature of the disease argues for monitoring of renal function with increasing age of patients, thus impacting patient management.

*Col4a1* mouse models led to the identification and characterization of mutations in humans and thus the description of clinical phenotypes. Based on this, it is likely that these phenotypes, including the medullary atrophy, diabetes insipidus, polyuria and urine-concentrating defect, proteinuria and mild hypotension, can develop in at least a proportion of these individuals with *COL4A1* mutations. The relative small number of families described to date, combined with a high level of clinical heterogeneity between and within families (Kuo et al., 2012; Vahedi and Alamowitch, 2011) and focus on the cerebrovascular disease aspects of *Col4a1* disease, can all explain why these phenotypes have yet to emerge and highlight the need for a further detailed clinical analysis of affected individuals. Our data to date strongly support a multifactorial origin of the hypotension in addition to the previously described defects in vascular function (Van Agtmael et al., 2010). Although our previous data suggested an association with the reduced blood volume in the presence of normal plasma volume (Van Agtmael et al., 2010), our Epo experiments described in this article directly establish this contribution. It is interesting in this regard that ER stress through the protein ATF4 can affect Epo production (Chiang et al., 2013). Moreover, we have identified a blunted response of the distal tubules to elevated aldosterone levels and increased compensatory sodium reabsorption in the proximal tubules in response to the hypotension. Given that the experiments with diuretics were performed on young animals, before the onset of medullary atrophy, this indicates a novel role for the basement membrane and the collagen IV network in the regulation of vascular homeostasis in distal tubules by being important for the response to aldosterone. These data show that a compensatory response occurs within the kidney to the reduced blood pressure by activation of RAAS but whereby the reduced response to aldosterone renders this insufficient to normalise blood pressure levels despite increased sodium reabsorption via proximal tubules. Furthermore, it is likely that altered vascular resistance is also an underlying factor, although this remains to be established.

Metabolic cage studies revealed that *Col4a1* mutations cause diabetes insipidus, because mice display the classical signs of reduced urine osmolality, polyuria and polydipsia. The diabetes insipidus in 2.5-month-old mice, before the onset of atrophy of the medulla, and reduced urine concentrating in response to vasopressin, suggests the development of diabetes insipidus with a central and nephrogenic origin. The development of chronic ER stress causing medullary atrophy with inability to elevate Aqp2 protein levels represents a molecular basis for the nephrogenic diabetes insipidus. The proteinuria is a consistent feature in *Col4a1* mutant mice, and our data indicate that this is not confined to the more severe mutations, such as glycine mutations and deletions (Favor et al., 2007; Gould et al., 2006). However, the origin of the proteinuria remains unclear. Although subtle GBM defects are likely to contribute and indicate the importance of this network for renal function, it has also been proposed that Bowman's capsule, which is severely affected in *Col4a1* renal disease, can act as a secondary barrier (Ohse et al., 2009). In addition, reduced tubular protein reabsorption (Nielsen and Christensen, 2010) and medullary atrophy could also contribute to the proteinuria. Although Bowman's capsule defects and haematuria are conserved between our mouse models and HANAC syndrome (Plaisier et al., 2007), we failed to detect any cyst formation or tubular BM defects. These phenotypic differences can reflect a separate disease mechanism, as it has been proposed that HANAC syndrome is caused by a distinct disease mechanism involving altered integrin signalling (Plaisier et al., 2010), supported by data published during the revision of the present paper (Chen et al., 2015). Moreover, our data show that genetic background also influences the development of *Col4a1* renal disease, which has also been observed for albuminuria (Long et al., 2013; Tsaih et al., 2009).

Many aspects of the pathomolecular mechanisms of *COL4A1* defects remain poorly characterised. Our data reveal that *Col4a1* mutations not only affect BM structure but that they also alter BM composition, including reduced incorporation of perlecan. This indicates that α1α1α2(IV) is required for perlecan deposition in the BM, which is supported by data from *Drosophila* (Pastor-Pareja and Xu, 2011). The ectopic expression of α3α4α5(IV) in Bowman's capsule represents a different mechanism compared with Alport syndrome, in which absence of α3α4α5(IV) can cause continued expression of α1α1α2(IV) in the GBM (Kalluri et al., 1997). To our knowledge, this is the first evidence of collagen IV network ‘switching’ induced by α1α1α2(IV) defects. The more cuboidal appearance of the epithelial cells (Fig. 4; also see Van Agtmael et al., 2005) and podocin expression suggest that this might reflect a cellular reprogramming, perhaps as a compensatory response. This is supported by cell reprogramming because of ER stress in chondrocytes with mutations in *Col10a1* (Tsang et al., 2007). Likewise, our analysis also revealed increased podocin expression in the glomerulus. Although reduced levels of podocin are well characterised in kidney disease (Mollet et al., 2009), the significance of increased expression remains unclear but could indicate a response to BM defects while also illustrating altered GBM composition. In conclusion, although their relative importance to the pathophysiology remains to be determined, defects in BM composition are part of the response to, and potentially mechanism of, *Col4a1* mutations.

A temporal analysis of ER stress and BM defects revealed that chronic ER stress appears to coincide with the development of tubular disease in the absence of BM defects. By contrast, 40-day-old animals present with BM defects and glomerulopathy, but much lower levels of chronic pathogenic ER stress compared with 3-month-old *Col4a1^+/Raw^* mice. This provides evidence for the occurrence of distinct pathomolecular mechanisms reflecting differences in the relative contribution of ER stress and BM defects to phenotype development. This has important consequences for the development of therapeutic avenues, because ER stress can be caused by mutations in other matrix proteins and be targeted by small molecules, including chemical chaperones (Engin and Hotamisligil, 2010; Murray et al., 2014). Rescue of BM defects and their associated phenotypes might require alternative approaches, including regeneration of functional intact BMs and secretion of accurately folded Col4a1. However, it now remains to be determined whether this disease mechanism applies to all *Col4a1* mutations, because disease mechanisms and renal phenotypes might be mutation dependent (Alamowitch et al., 2009; Kuo et al., 2014; Plaisier et al., 2010, 2007; Van Agtmael et al., 2005).

In conclusion, *Col4a1* mutations cause renal disease that involves cell-specific disease mechanisms, including ER stress and BM defects. These data will inform the development of therapeutic strategies for renal diseases resulting from collagen mutations.

## MATERIALS AND METHODS

### Animal studies

All animal studies were performed in accordance with the UK Home Office under Project licence 60/4132.

### Electron microscopic analysis

Tissue were collected and fixed in 2% glutaraldehyde in phosphate buffer and processed as previously described (Taylor et al., 2011). EM thickness was measured using ImageJ (http://imagej.nih.gov/ij/), whereby thickness was measured on EM images (1900× magnification) using a grid with a measure being taken every ∼800 nm (total of ∼90 measurements).

### Histopathology

Kidneys were fixed in 10% neutral buffered formalin or 4% paraformaldehyde and embedded in paraffin wax. Sections were stained with Haematoxylin and Eosin, PicroSirius Red or Perls’ Prussian Blue using standard protocols.

### Systolic blood pressure analysis

Systolic blood pressure was measured using tail-cuff plethysmography as previously described (Van Agtmael et al., 2010).

### *In vivo* renal function studies

*In vivo* renal function studies were performed as described by Craigie et al. (2012). Under anaesthesia, mice were infused intravenously with isotonic saline at a rate of 200 µl/h/10 g body weight. After a 40 min equilibration period, mean arterial blood pressure was recorded for 30-40 min and urine was collected for 1 h. Evans Blue (1 µl/g of a 0.5% solution w:v) was injected for measurement of plasma volume; blood volume was calculated from this and haematocrit. After baseline collections, each mouse received an intravenous injection of furosemide (3 mg/kg), thiazide (2 mg/kg) or amiloride (2 mg/kg). The natriuretic response to these agents was assessed as an index of the *in vivo* activity of the Na^+^-K^+^-2Cl^−^ cotransporter (NKCC2), the Na^+^-Cl^−^ cotransporter (NCC) and the epithelial sodium channel (ENaC), respectively (Bailey et al., 2009; Hunter et al., 2014, 2015). At the end of the experiment, a 500 µl blood sample was taken for measurements of plasma sodium and potassium. Sodium and potassium concentration in urine and plasma was measured by ISE (Roche 9180, UK).

### Metabolic cage studies

Animals were individually housed and allowed to acclimatise for 24 h, after which daily urine samples were collected and water consumption was measured. Water was withdrawn for 24 h. Vasopressin (Sigma) administration occurred via intraperitoneal injection, and animals were subsequently housed in metabolic cages for 6 h. Urine samples were collected 6 h after intraperitoneal injection and via bladder massage/vasopressin. Six microlitres of urine was loaded on an SDS-PAGE gel, followed by silver staining for analysis of proteinuria, whereas ELISA was used to measure albumin-to-creatinine ratios.

### Immunoblotting

Protein extracts were prepared in RIPA buffer containing EDTA protease (Roche Applied Science) and phosphatase inhibitors (Phostop Roche). Membranes were blocked with 5% milk or bovine serum albumin before incubation with primary antibodies BIP (1:10,000; BD Transduction 610979), ATF4 (1:2500; Santa Cruz Sc-200), NHE3 (Stressmarq SPC-400D; 1:1000), p-NHE3 (Santa Cruz Sc-53961; 1:200), AQP2 (Santa Cruz Sc-9882; 1:800), NKCC2 (1:1000) and CHOP (Santa Cruz). Membranes were incubated with horseradish peroxidase-conjugated secondary antibodies (GE Healthcare) and developed using chemiluminescense (Millipore). For cleaved caspase 3 (Cell Signaling Technology; 1:1000), primary and secondary antibodies (LI-COR) were incubated using 50% Seablock bluffer (ThermoFisher) and 50% tris-buffered saline-0.1% tween (TBST), and signals were detected using Odyssey Sa imager (LI-COR). Protein levels were corrected for Coomassie staining of total protein gels run in parallel with the western blot gels or by total protein stain on the membrane (Memcode; Pierce). Densitometry was performed using ImageJ software. Statistical analysis (Graphpad Prism) was done using Student's unpaired *t*-test.

### Immunohistochemistry

Immunohistochemistry was performed as described by Taylor et al. (2011). After fixation in acetone and antigen retrieval, sections were incubated with primary antibodies [H11 for Col4a1, H22 for Col4a2 and H31 for Col4a3 (all 1:100), perlecan (1:1000), nidogen 1 (1:1000), nidogen 2 (1:1000), Col1a1 (Abcam Ab292; 1:1000), Bip (Abcam; 1:1000) and laminin (Sigma; 1:50)] overnight at 4°C. Incubation with secondary antibodies (1:400; Jackson ImmunoResearch) was performed and slides were mounted using Vectashield (Vector). Images were captured using a non-inverted fluorescent microscope (Zeiss Axioskop-Axiocam) and captured through AxioVision 4.8 (Zeiss) or as *z*-stacks using a Zeiss LSM510 Meta confocal microscope and Zeiss LSM software (Zeiss, Germany), which was also used for generating projections. ImageJ analysis was used for quantification of fluorescence as previously described (Murray et al., 2014).

### Haematological analysis

Epo was administered via intraperitoneal injection of 150 units (three times per week) of human recombinant erythropoietin (gift from Dr Jo Mountford, University of Glasgow).

### Statistical analysis

Statistical analysis (Graphpad Prism) was done by Student's unpaired *t*-test and by ANOVA and Bonferroni post hoc test for *in vivo* renal function assays.
